# Structural geometries and mechanical properties of vertebral implant with honeycomb sandwich structure for vertebral compression fractures: a finite element analysis

**DOI:** 10.1186/s12938-021-00934-z

**Published:** 2021-10-02

**Authors:** Yuan Guo, Jing Liu, Xushu Zhang, Zejun Xing, Weiyi Chen, Di Huang

**Affiliations:** 1 College of Biomedical Engineering, Taiyuan University of Technology, Taiyuan, 030024 China; 2 Shanxi Bethune Hospital, Shanxi Academy of Medical Sciences, Taiyuan, 030032 China

**Keywords:** Honeycomb sandwich structure, Vertebral body implant, Vertebral compression fracture, Mechanical properties, Orthogonal test method, Finite element analysis

## Abstract

**Background:**

Because of osteoporosis, traffic accidents, falling from high places, and other reasons, the vertebral body can be compressed and even collapse. Vertebral implants can be used for clinical treatment. Because of the advantages of honeycomb sandwich structures, such as low cost, less material, light weight, high strength, and good cushioning performance. In this paper, the honeycomb sandwich structure was used as the basic structure of vertebral implants.

**Methods:**

The orthogonal experiment method is applied to analyse the size effect of honeycomb sandwich structures by the finite element method. Based on the minimum requirements of three indexes of peak stress, axial deformation, and anterior–posterior deformation, the optimal structure size was determined. Furthermore, through local optimization of the overall structure of the implant, a better honeycomb sandwich structure vertebral implant was designed.

**Results:**

The optimal structure size combination was determined as a panel thickness of 1 mm, wall thickness if 0.49 mm, cell side length of 1 mm, and height of 6 mm. Through local optimization, the peak stress was further reduced, the overall stress distribution was uniform, and the deformation was reduced. The optimized peak stress decreased to 1.041 MPa, the axial deformation was 0.1110%, and the anterior–posterior deformation was 0.0145%. A vertebral implant with good mechanical performance was designed.

**Conclusions:**

This paper is the first to investigate vertebral implants with honeycomb sandwich structures. The design and analysis of the vertebral implant with a honeycomb sandwich structure were processed by the finite element method. This research can provide a feasible way to analyse and design clinical implants based on biomechanical principles.

## Background

In recent years, the frequent occurrence of diseases, natural disasters, traffic, the ageing of the population, obesity, lack of exercise and other external factors have led to a growing amount of clinical bone tissue damage, and clinical demand for bone defect repair is growing [1]. Bone has a strong self-repair ability when the injury is minor. However, for bone defects beyond the critical size, implants are needed to repair bone defects, including autogenous and allogeneic or artificial bone grafts [2]. Autologous bone grafts have better adaptability for bone grafting. However, because of limited donor bone grafts and the incidence rate of allograft or immune rejection, it is impossible to widely apply [2]^.^ Researchers have turned their attention to artificial bone repair materials [3, 4].

Generally, artificial bone repair materials must have good compatibility with surrounding cell tissue to promote the repair and healing of the defect site. In addition, implants must also have good mechanical properties to withstand the load during the bone defect repair process and to provide a stable and complete structure [1, 4]. However, these artificial bone materials are limited to the critical-sized defects of non-bearing bones due to their poor mechanical properties [4, 5]. Many methods can be used to improve the mechanical properties of the implant, for example, by compounding other materials while optimizing the structure of the implant. According to the structural characteristics and excellent mechanical properties of the honeycomb, a porous chitosan and nano-hydroxyapatite composite scaffold (CS/nHA) was manufactured with 3D printing by Hongxia Zhao [6]. The scaffold with high porosity was found to improve compressive strength (1.62 ± 0.22 MPa) and Young’s modulus (110 ± 22 MPa), which was similar to that of cancellous bone. According to the osteogenic capacity and mechanical properties, including the excellent strength and toughness of natural bone, HA/collagen composite nanofibres were used to construct a kind of bone scaffold with a bionic multilayer hierarchical structure similar to natural bone components by Bian et al. [7]. The compressive strength of the multistage hierarchical bone structure scaffold was 3 MPa.

Porous scaffolds with honeycomb structures, which exhibit excellent mechanical properties, have great application potential in tissue engineering [8]. A honeycomb sandwich is a structure that consists of two relatively thin panels bonded to a relatively thick lightweight honeycomb core [9]. The panels primarily carry tensile and compressive loads that have high stiffness and strength, and the core has sufficient shear strength to withstand transverse shear stresses and is thick to provide high shear stiffness to resist buckling of the panel [10]. Structures are broadly used in automotive, aerospace, and transportation and many other fields because of their high strength, high bending stiffness/weight ratio, light weight, and so on [11]. Therefore, this kind of structure with good mechanical properties is also needed in the field of clinical bone implantation. It can not only fill bone defects and restore the geometric size of the original bone tissue, but also meet the biomechanical requirements of the bone tissue.

The spine is an important part of the human body's system, which functions to support the trunk and protect the internal organs and the spinal cord. It is also a place that is relatively easy to injure. Common forms of spinal load are tension, bending, torsion, shear composite load, and mainly compression load. Because of osteoporosis, traffic accidents, falling from high places and other reasons, the vertebral body could suffer compressed fracture or even collapse [12]. 1.4 million vertebral compression fractures occur every year in the world. A compression fracture is a fracture of the vertebrae that results in a reduction in the height of the vertebrae by at least 20%. Vertebral compression fractures often occur at the midthoracic (T7–T8) spine and the thoracolumbar junction (T12–L1) [13, 14]. The clinical treatment of vertebral compression fractures includes conservative treatment (such as physical therapy and spinal orthosis), vertebroplasty (such as minimally invasive percutaneous vertebroplasty and kyphoplasty) and vertebral implantation (such as SpineJack® and Vertebral Body Stent®) [15]. In clinical treatment of vertebroplasty, bone cement is commonly used. Bone cement filling material has no biological activity and cannot be accurately designed [16]. The operation mainly depends on experience, and has certain toxic and side effects, which may affect the requirements of normal stress distribution of adjacent tissues.

In the initial stage of clinical treatment of vertebral implants, because of good adaptability, autologous bone and allogeneic bone transplantation were used. However, due to the difficult source of autologous bone, and the low safety of allogeneic bone or the impact of rejection, they cannot be widely used. At present, the vertebral implants commonly used in the clinic are metal vertebral bodies or filling materials similar to vertebral body implants. Metal vertebral implant, for example titanium and its alloys, have been successfully applied in orthopaedic implants because of their excellent mechanical properties. These titanium and titanium alloys often cause stress shielding due to mismatch of Young’s modulus between the bio-titanium (110 GPa) and the surrounding bone (10–30 GPa) [17]. This mismatch can result in bone absorption and eventually the implant loosens clinical performance in a long term when it is implanted into the bone host [18, 19]. Honeycomb sandwich vertebral implants have the advantages of high design accuracy, light weight, stable structure and excellent mechanical properties.

Vertebral implants with good mechanical properties have uniform pressure distribution, no stress concentration, good stability and small strain in all directions. Honeycomb structures with different geometric sizes have different mechanical properties, so optimizing the geometric parameters of the structure is an important means to improve its mechanical properties [9–11]. The "orthogonal test method" is a design method with multiple factors and multiple levels [20]. According to the four factors that affect the mechanical properties of the honeycomb sandwich structure, the orthogonal test was designed with four factors and three levels. In addition, the ratio of geometric parameters can also be defined as one of the influencing factors to effectively analyse the size effect on the mechanical properties of vertebral implants.

In this paper, the size effect of the honeycomb sandwich structure was analysed using the finite element analysis software ABAQUS and the orthogonal test method. The optimal structure size was determined when three indexes of peak stress, axial deformation, and anterior–posterior (AP) deformation of the structure were the minimum simultaneously. In addition, through the local optimization of the structure, a honeycomb sandwich structure vertebral implant with a stable structure and good mechanical performance was designed. The influence of the ratio of the geometric parameters on the mechanical properties of the structure was analysed in this paper, and it is helpful to determine the range of geometric parameters in the design of vertebral implants with honeycomb sandwich structures.

## Results

The orthogonal test was designed with four factors and three levels. Four factors and three levels were set as panel thickness (factor A), and the values were 0.8 mm (1), 0.9 mm (2), and 1 mm (3); cell wall thickness (factor B), which was 0.28 mm (1), 0.49 (2) mm, and 0.70 (3) mm; cell side length (factor C), which was 1 mm (1), 2 mm (2), and 3 mm (3); and cell height (factor D), which was 6 mm (1), 9 mm (2), and 12 mm (3).

The orthogonal experimental results of nine different combinations of structural geometric parameters are shown in Table 1. The size effect of the honeycomb sandwich structure on the three indexes of peak stress, axial deformation, and AP deformation could be obtained.

**Table 1:** Result of the orthogonal experiment

	Factors				Results		
	A	B	C	D	Peak stress (MPa)	Axial deformation (%)	AP deformation (%)
1	1	1	1	1	1.354	0.1974	0.0277
2	1	2	2	2	3.407	0.3943	0.0593
3	1	3	3	3	6.692	0.7831	0.0993
4	2	1	2	3	3.511	0.3210	0.0647
5	2	2	3	1	5.979	1.0679	0.1125
6	2	3	1	2	1.160	0.1935	0.0294
7	3	1	3	2	6.165	0.7518	0.1227
8	3	2	1	3	1.243	0.1521	0.0258
9	3	3	2	1	2.881	0.3563	0.0382

Taking the peak stress, axial deformation and AP deformation of the structure as indexes, the average value of the experimental results at each level was calculated, which was recorded as *K*. The optimal level of each factor was judged according to the value of *K*, and the optimal level of each factor was taken as the structural combination parameter. The difference between the maximum value and the minimum value of *K* was calculated, which was recorded as the range *R*. According to the size of *R*, the order of the influence of each factor on the index was judged [21]. The range analysis of orthogonal experimental results is shown in Table 2. The size effect of the honeycomb sandwich structure was analysed. According to the minimum requirements of each indicator, the optimal structural combination parameters were selected.

**Table 2:** Range analysis of the orthogonal experiment

	Peak stress (MPa)				Axial deformation (%)				AP deformation (%)			
	A	B	C	D	A	B	C	D	A	B	C	D
*K*1	3.818	3.677	1.252	3.405	0.4583	0.4234	0.1810	0.5405	0.0621	0.0717	0.0276	0.0595
*K*2	3.550	3.543	3.267	3.577	0.5275	0.5381	0.3572	0.4466	0.0688	0.0658	0.0540	0.0705
*K*3	3.430	3.578	6.279	3.815	0.4201	0.4443	0.8676	0.4187	0.0622	0.0556	0.1115	0.0632
*R*	0.388	0.134	5.027	0.410	0.1074	0.1147	0.6866	0.1218	0.0068	0.0161	0.0839	0.0110

Table 2 shows that for the peak stress index, the *K*3 value of factor A was less than other values, which indicates that when factor A was at level 3, the peak stress was lower than other levels; similarly, the *K*2 value of factor B was close to *K*3 and less than other values. *For factors C and D,* the *K*1 value was the minimum, so level 1 was taken as the optimal level. Considering the peak stress, the optimal geometric parameter combination of the honeycomb sandwich structure *was A3 B2/3 C1 D1. For* the range *R*, RC > RD > RA > RB, which was the factor affecting the peak stress from the primary to the secondary order, were the cell side length, cell height, panel thickness, and cell wall thickness.

Similarly, for the axial deformation index, the optimal geometric parameter combination *was A3 B1 C1 D3. For* the range *R*, RC > RD > RB > RA, that is, the factors affecting the axial deformation index from the primary to the secondary order were cell side length, cell height, cell wall thickness, and panel thickness. For the AP deformation index, the optimal geometric parameter combination was A1/3 B3 C1 D1. For the range *R*, RC > RB > RD > RA; that is, the factors affecting the AP deformation index from the primary to the secondary order were cell side length, cell wall thickness, cell height, and panel thickness.

Based on the above analysis, for the three indexes of peak stress, axial deformation, and AP deformation, the optimal values of factors A, and C were all A3 C1. For factors D, the optimal values of peak stress and anterior–posterior (AP) deformation was D1, and for the index of axial deformation, there is little difference between D1 and D3, so the optimal values of factors A, C, and D were A3 C1 D1. But the optimal level of factor B was inconsistent. Therefore, when the other three factors were selected as the optimal level combination, a single-factor five-level test analysis of factor B was conducted. The results are shown in Table 3.

**Table 3:** Single-factor five-level test results of cell wall thickness

Factor B	Indexes		
	Peak stress (MPa)	Axial deformation (%)	AP deformation (%)
0.28	1.246	0.1788	0.0245
0.385	1.260	0.1725	0.0259
0.49	1.141	0.1688	0.0258
0.595	1.246	0.1663	0.0270
0.7	1.238	0.1638	0.0281

**Fig. 1:**
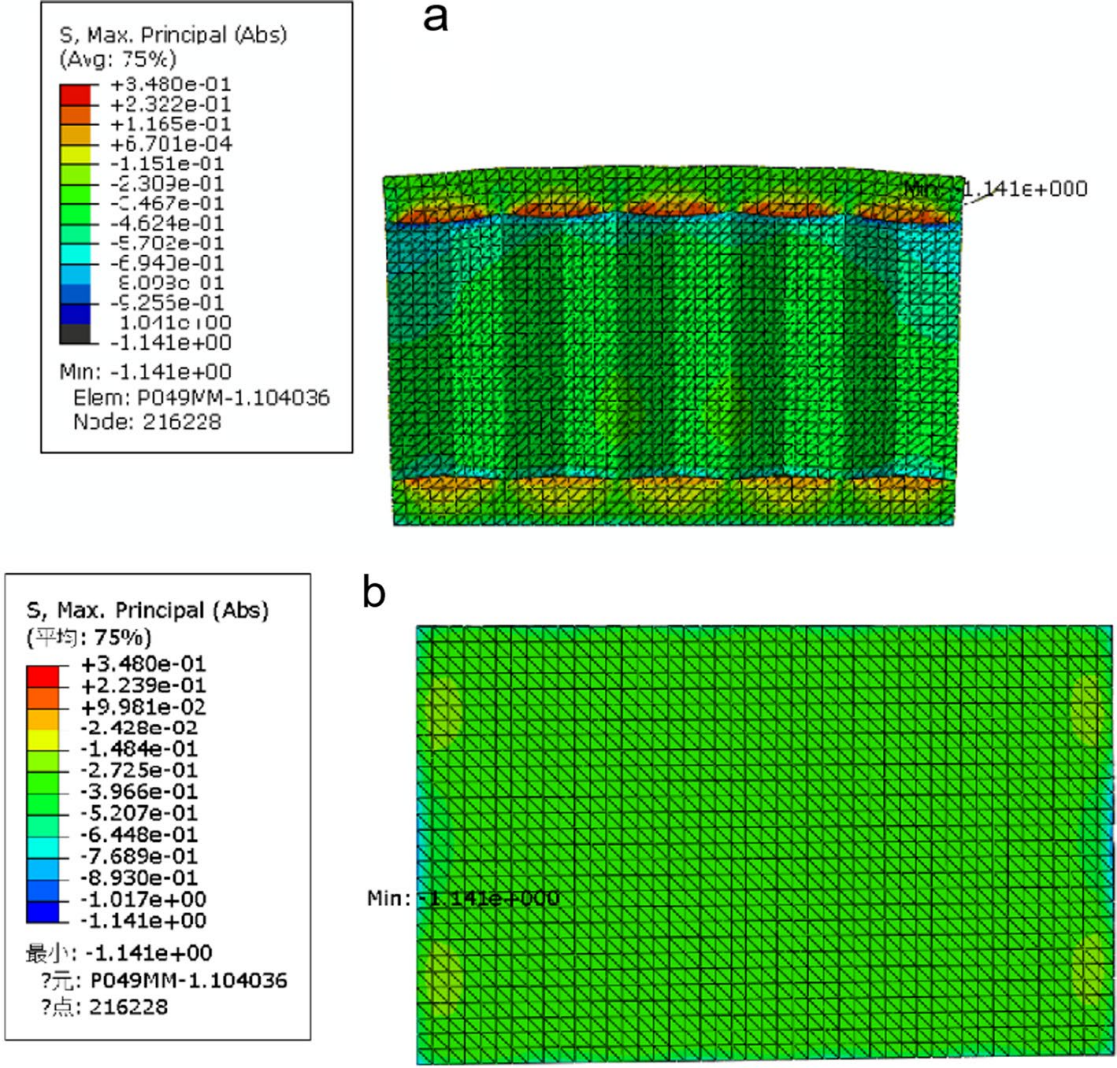
Stress distribution diagram of honeycomb sandwich structure: **a** the whole structure, **b** lower panel

Table 3 shows that when the other three factors were the optimal combination, the peak stress reached the minimum when the cell wall thickness was 0.49 mm. With increasing cell wall thickness, the axial deformation values decreased, and the AP deformation values increased. Considering the three indexes, a wall thickness of 0.49 mm was selected as the optimal value. Through the above analysis, it could be concluded that the optimal parameter combination of the honeycomb sandwich structure was A3 B2 C1 D1. The optimal combination of structural geometric parameters of the honeycomb sandwich structure was determined as follows: panel thickness, cell wall thickness, cell side length, and cell height were 1 mm, 0.49 mm, 1 mm, and 6 mm, respectively. The stress distribution diagram of the honeycomb sandwich structure under compression is shown in Fig. 1. The peak stress was 1.141 MPa, and the deformation was calculated according to formula 1. The axial deformation was 0.1688%, and the AP deformation was 0.0258%:

1 $$\mathrm{deformation}\left(\%\right)=\frac{\mathrm{deformation \, displacement}}{\mathrm{total \, length}}\times 100\%.$$

**Fig. 2:**
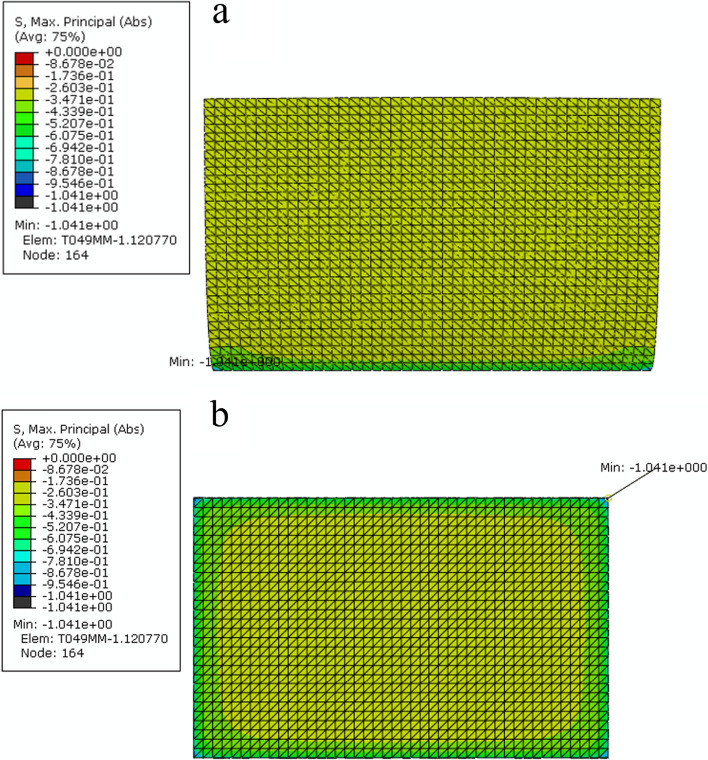
Stress distribution diagram of honeycomb sandwich structure with local optimization: **a** the whole structure, **b** lower panel

Furthermore, the honeycomb sandwich structure was locally optimized, and the structure of the surrounding edge was implemented to reduce deformation. After loading the same load, the stress distribution diagram is shown in Fig. 2.

Figure 2 shows that after local optimization of the honeycomb sandwich structure, the internal stress distribution was uniform, and the maximum peak stress was 1.041 MPa, which was lower than that before the structure. This result indicated that the peak stress of the structure was reduced through local optimization. The peak position occurred on the edge of the lower panel. The overall axial deformation was 0.1110%, and the AP deformation was 0.0145%, which were both less than those before the optimization, showing that the deformation degree of the honeycomb sandwich structure decreased after local optimization. Large deformation and stress concentrations can be effectively avoided through local optimization, and the safety of the implant is ensured. Finally, a honeycomb sandwich structure vertebral implant with a stable structure and high mechanical performance is designed. The optimal overall size of this implant is 11.11 mm long, 6.98 mm wide and 8 mm high.

## Discussion

Due to the geometric complexity of the honeycomb structure, the geometric structure can be simplified to clarify the relationship between the equivalent elastic parameters and geometric parameters, and then equivalent elastic parameters are used to describe the mechanical properties of the honeycomb structure [22]. Commonly used geometric parameters are cell wall thickness *t*, cell side length *l*, and cell height *h*. The size effect of the honeycomb structure is divided into an in-plane size effect and an out-of-plane size effect. The in-plane size effect refers to the influence of changes in cellular in-plane size on the mechanical properties of the sandwich structure, such as the cell wall thickness and the cell side length. The out-of-plane size effect refers to the effect of changes in the cell height direction size on the mechanical properties of the sandwich structure.

### Influence of the ratio of wall thickness to cell side length on mechanical indexes

Based on Gibson’s formula [23], the formula of the equivalent elastic modulus of the honeycomb material was reduced by Fu Minghui [24]. For the regular hexagonal honeycomb structure, each equivalent elasticity parameter was only related to the ratio of wall thickness to cell side length of the honeycomb. When the panel thickness and height were the optimal geometric parameters, the influence of the ratio of the wall thickness to the cell side length (*t/l*) on the mechanical properties of the honeycomb sandwich structure was analysed, as shown in Table 4.

**Table 4:** The influence of *t/l* on mechanical indexes

Wall thickness (*t*)	Cell side length (*l*)	*t/l*	Peak stress (MPa)	Axial deformation (%)	AP deformation (%)
0.49	3	0.163	5.576	0.8875	0.2642
0.49	2	0.245	2.908	0.3763	0.0759
0.28	1	0.28	1.246	0.1788	0.0192
0.49	1	0.49	1.141	0.1688	0.0150
0.7	1	0.7	1.238	0.1638	0.0123

Table 4 shows that with the increase in the ratio of wall thickness to cell side length (*t/l*), the peak stress first decreased and then increased, the axial deformation values and the AP deformation values decreased. The results showed that the mechanical properties of the honeycomb sandwich structure had an obvious in-plane size effect. Comprehensively, a *t/l* of 0.49 was the optimal value, which was consistent with the optimal geometric parameter combination obtained above.

### Influence of the ratio of cell side length to height on mechanical indexes

Ma Lianhua et al. [25] proved that the equivalent elastic parameters of honeycomb sandwich structures are related not only to the structural parameters of the cell, but also to the sandwich height. The experimental results of Pan et al. [26] and Wang Dongmei et al. [27] showed that the influence of the cell height on the shear modulus and strength of honeycomb sandwiches was significant. When the panel thickness and the cell wall thickness were optimal geometric parameters, the influence of the ratio of the cell side length to the height (*l/h*) on the mechanical properties of the honeycomb sandwich structure was analysed, as shown in Table 5.

**Table 5:** The influence of *l/h* on mechanical indexes

Cell side length (*l*)	height (*h*)	*l/h*	Peak stress (MPa)	Axial deformation (%)	AP deformation (%)
1	12	0.083	1.243	0.1521	0.0258
1	9	0.111	1.237	0.1582	0.0258
1	6	0.167	1.141	0.1688	0.0258
2	6	0.333	2.908	0.3763	0.0442
3	6	0.5	5.576	0.8875	0.0909

Table 5 shows that with increasing *l/h*, the peak stress values first decreased and then increased, the axial deformation values and the AP deformation values slowly increased. The results showed that the mechanical properties of the honeycomb sandwich structure had an obvious out-of-plane size effect. Comprehensively, the ratio of cell side length to height (*l/h*) of 0.167 was a better value, which was consistent with the optimal geometric parameter combination obtained above.

By analysing the influence of the ratio of cell wall thickness to cell side length and cell side length to height on the mechanical properties of the structure, it is helpful to determine the range of geometric parameters in the design of honeycomb sandwich structure implants.

At present, the vertebral implant used for spinal fracture is percutaneous inserted into the posterior part of the vertebral body, and is expanded to reduce the fracture and restore the anatomy. Take SpineJack® and Vertebral Body Stent ® as examples. The former is a titanium device for third generation percutaneous vertebral augmentation procedures (PVAPs). SpineJack is inserted into the vertebral body to expand the compressed vertebral body, which can be divided into cemented and cementless. The latter is a balloon-expandable, barrel-shaped, metallic device, which is inserted via monopedicular or bipedicular access. On expansion, Vertebral Body Stent® (VBS) keeps the created cavity open after balloon deflation until cement is injected. As mentioned, the main function of SpineJack® and Vertebral Body Stent ® are to recovery the anatomy height, and the biomechanical requirements of vertebral compression injury are not considered in the design. In this paper, the optimal structure size and biomechanical properties of honeycomb sandwich structure vertebral implant were analysed with finite element method; a stable structure with good mechanical performance was designed before operation.

The design and analysis of the vertebral implant with a honeycomb sandwich structure were processed by the finite element method. This research can provide a feasible way to analyse and design clinical implants based on biomechanical principles. It can provide customized and personalized implants for patients. In this paper, the load of 400 N on the top surface applied on the model was based on the lumbar load of a healthy adult. Although for the applied load, the peak stress and deformation obtained may be different with the ages and weights of patients, the research methods and conclusions of this article are generally applicable for implant design. In this paper, the honeycomb unit cell shape is a regular hexahedron with good mechanical properties. Different unit cell shapes can also be designed to obtain a honeycomb sandwich structure with better mechanical properties in future work.

## Conclusions

In this paper, the honeycomb sandwich structure is first used as the basic structure of the vertebral implant. Combined with the orthogonal test method, finite element analysis is performed on the size effect of the honeycomb sandwich structure. Based on the minimum requirements of the peak stress, axial deformation, and AP deformation, the optimal geometric parameters were determined. Through the local optimization of the implant structure, a honeycomb sandwich vertebral implant with a stable structure and good mechanical properties was designed. Honeycomb sandwich structure vertebral implants provide personalized precision design, and the design conforms to vertebral biomechanics. Because the implant is to be placed inside the vertebral body, the cortical bone of the vertebral body should be relatively complete, which is not suitable for large-scale bone defects.

1. According to the range analysis of the orthogonal test, the factors that affect the peak stress index from primary to secondary order are the cell side length, cell height, panel thickness, and cell wall thickness. The factors that affect the axial deformation index from primary to secondary order are the cell side length, cell height, cell wall thickness, and panel thickness. The factors that affect the AP deformation index from primary to secondary order are cell side length, wall thickness, cell height, and sheet thickness.
2. The optimal structure size was determined when the minimum requirements of three indexes of peak stress, axial deformation, and anterior–posterior (AP) deformation of the structure were the minimum simultaneously: the panels thickness 1 mm, wall thickness 0.49 mm, cell side length 1 mm, and height 6 mm. According to this size combination, a honeycomb sandwich structure with good mechanical properties is designed. The peak stress was 1.141 MPa, the axial deformation was 0.1688%, and the AP deformation was 0.0258%.
3. In addition, through local optimization of the structure, the peak stress is further reduced, the overall stress distribution is uniform, and the deformation is reduced. The optimized peak stress decreased to 1.041 MPa, the axial deformation was 0.1110%, and the AP deformation was 0.0145%.
4. Through analysis of the ratio of the cell wall thickness to the cell side length (*t/l*) and the cell side length to the height (*l/h*), it is found that the mechanical properties of the honeycomb structure have obvious in-plane and out-of-plane size effects. When *t/l* is 0.49 and *l/h* is 0.167, the three mechanical indexes are improved. The result was consistent with the optimal geometric parameter combination obtained above.

Compression load is the main form of vertebral bearing, and this article only analyses the compression force of the honeycomb sandwich structure. The research idea and method of this paper can be applied to the design of vertebral implants under various load forms. In the future, the design of structural parameters of vertebral implants under other load forms will be carried out.

## Methods

In this paper, the size effect of the honeycomb sandwich structure was analysed using the orthogonal test method. The orthogonal test method is a design method with multiple factors and multiple levels. A group of orthogonal tables was used to design the experimental scheme, and the experimental results were analysed. According to orthogonality, several representative test conditions are selected, and the best or better scheme is found through these test data. It is an efficient, rapid, and economic experimental design method [28]. The honeycomb sandwich structures with different geometric sizes have different mechanical properties; the geometric size of the structure is optimized by orthogonal test to improve its mechanical properties.

**Fig. 3:**
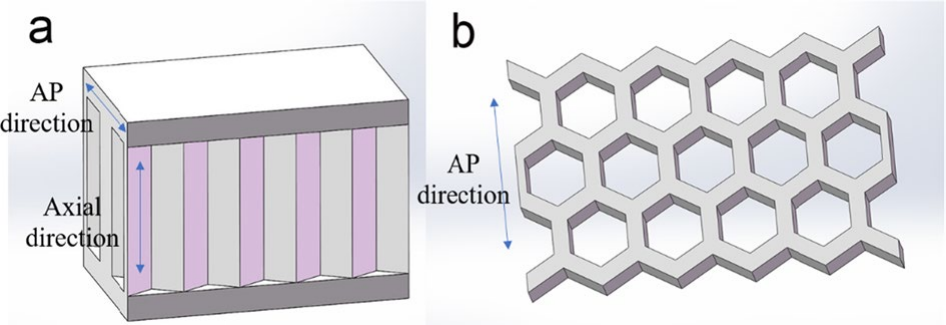
The model of honeycomb sandwich structure: **a** honeycomb sandwich, **b** honeycomb core

**Fig. 4:**
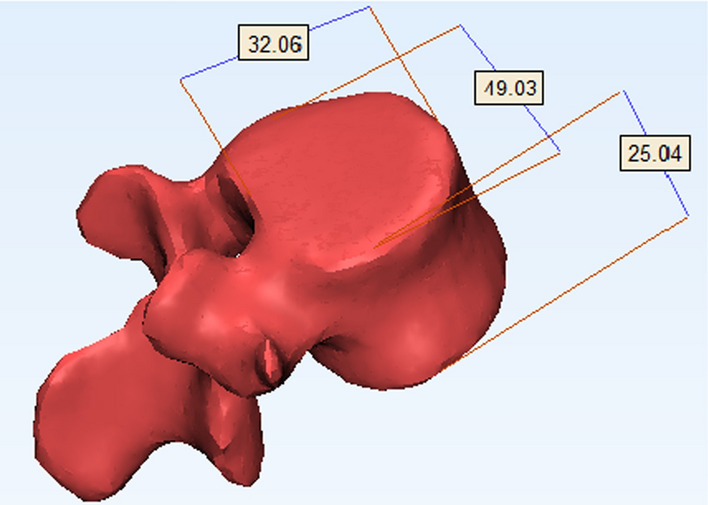
Geometric model of human lumbar spine (L4) (mm)

The geometric model of the 5 × 3 honeycomb sandwich was established using SolidWorks software (Dassault Systemes, Trial version). The model was composed of upper, lower panels and honeycomb core, as shown in Fig. 3. The above data in Tables 1, 2, 3, 4, 5 are derived from the model in Fig. 3. Clinically, posterior surgery is used to treat vertebral body compression fractures. Therefore, the designed implant should be smaller than the anatomical size of the vertebral body and the incision size of the posterior surgery. The selected factors and levels not only meet the requirements of lumbar anatomy and clinical surgery, but also reflect the influence trend of the size effect. A healthy adult male volunteer was selected as the test object, without lumbar disease and injury. The biomechanical properties of the 4th lumbar vertebra were given that were similar to cancellous bone. The elastic modulus was 291 MPa, the Poisson's ratio was 0.25, the density was 0.17e^−06^ kg/mm^3^, yield stress was 1.92 MPa, and failure strain was 14.5e^−03^ [29]. A 3D geometric model of L4 based on its CT scan data is shown in Fig. 4. Its height is 25.04 mm, mediolateral width is 49.03 mm, AP length is 32.06 mm.

Four factors and three levels were set as panel thickness (factor A), with values of 0.8 mm, 0.9 mm, and 1 mm; cell wall thickness (factor B), with values of 0.28 mm, 0.49 mm, and 0.70 mm; cell side length (factor C), with values of 1 mm, 2 mm, and 3 mm; and cell height (factor D), with values of 6 mm, 9 mm, and 12 mm.

A three-dimensional finite element model of a honeycomb sandwich structure implant was established using the finite element software ABAQUS 6.11. The element type was tetrahedral element C3D10, and the element size of the whole model was 0.25 mm. The implant material property was assumed to be an isotropic linear elastic material, and the mechanical parameters of the material were the same as those of cancellous bone. The elastic modulus was 291 MPa, the Poisson's ratio was 0.25, and the density was 0.17e^−06^ kg/mm^3^ [29] The bottom panel of the honeycomb sandwich structure was fixed and constrained, and the load on the upper panel was applied to simulate the load on the upper surface of the L4 lumbar spine when standing on two feet; that is, the upper body weight of the human body was 400 N [30]. The 400 N load on the top surface applied on the model is based on the lumbar load of a healthy adult male, in which the axial compression condition of the honeycomb sandwich structure implant was simulated.

Nine different combinations of structural geometric parameters were simulated by the finite element method, and three indexes of peak stress, axial deformation, and anterior–posterior deformation (AP deformation) of each parameter combination were recorded. According to the minimum requirement, under the same load, the smaller the peak stress is, the better. This shows that the implant has a better bearing capacity and effectively avoids stress concentrations. Therefore, when three indexes were minimized simultaneously, the optimal geometric parameters were determined.

**Fig. 5:**
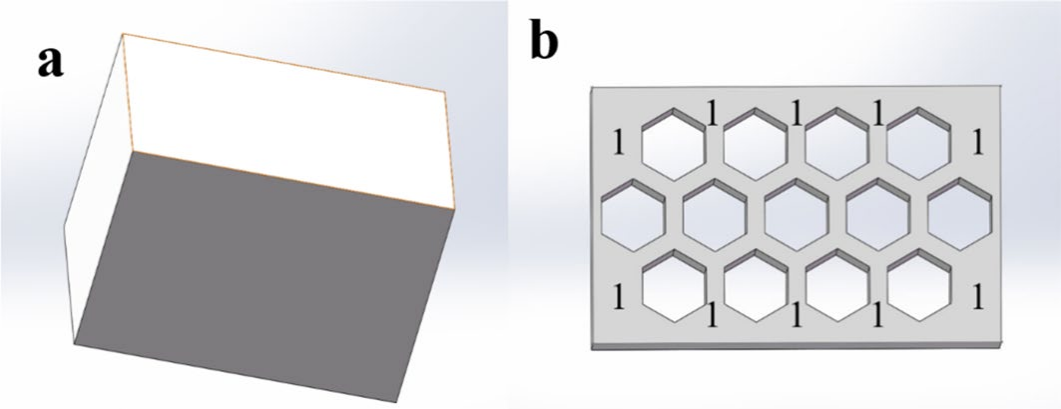
Model of honeycomb sandwich structure with local optimization: **a** the whole structure, **b** honeycomb core

The stress distribution diagram (Fig. 1) of the honeycomb sandwich structure is obtained above. The peak stress was concentrated at the edge of the honeycomb sandwich structure without support. Because the implant design needs to meet the function of supporting the vertebral body and restoring the original height of the vertebral body, the deformation of the implant after loading should be as small as possible. To avoid large deformation and reduce stress concentration as much as possible, the honeycomb sandwich structure was locally optimized, and the structure of the surrounding edge area lacking support was supplemented (Fig. 5). A honeycomb sandwich structure vertebral implant with a stable structure and high mechanical performance was designed. In this paper, a method of personalized customized vertebral implant was proposed. According to the patient's vertebral body injury, through full communication with the surgeon, the geometric size range of honeycomb sandwich structure implant was given. The optimal structure size was determined by orthogonal test, then by supplementing the sandwich structure surrounding edge area, and the vertebral implant was determined, in order to reduce the fracture, restore the anatomy and meet the requirements of long-term biomechanics. At the same time, implants using the 3D printing method can be easily obtained. In the specific operation implementation, based on the current percutaneous operation mode, combined with the injury situation and the size of vertebral implants, the appropriate operation mode would be determined by surgeons.

## Data Availability

Not applicable.

## Abbreviation

AP: Anterior–posterior
